# GABAergic interneurons contribute to the fatal seizure phenotype of CLN2 disease mice

**DOI:** 10.1172/jci.insight.184487

**Published:** 2025-08-21

**Authors:** Keigo Takahashi, Nicholas R. Rensing, Elizabeth M. Eultgen, Letitia L. Williams, Sophie H. Wang, Hemanth R. Nelvagal, Steven Q. Le, Marie S. Roberts, Balraj Doray, Edward B. Han, Patricia I. Dickson, Michael Wong, Mark S. Sands, Jonathan D. Cooper

**Affiliations:** 1 Department of Pediatrics, and; 2 Department of Neurology, Washington University in St. Louis, St. Louis, Missouri, USA.; 3 Department of Pharmacology, School of Pharmacy, University College London, London, United Kingdom.; 4 Department of Medicine,; 5 Department of Neuroscience, and; 6 Department of Genetics, Washington University in St. Louis, St. Louis, Missouri, USA.

**Keywords:** Genetics, Neuroscience, Genetic diseases, Lysosomes, Seizures

## Abstract

The cellular etiology of seizures in CLN2 disease, a childhood-onset neurodegenerative lysosomal storage disorder caused by a deficiency of tripeptidyl peptidase 1 (TPP1), remains elusive. Given that *Cln2^R207X/R207X^* mice display fatal spontaneous seizures and an early loss of several cortical GABAergic interneuron populations, we hypothesized that these 2 events might be causally related. To study the cell-autonomous effects of interneuron-specific TPP1 deficiency, we first generated transgenic mice expressing *loxP*-flanked lysosomal membrane–tethered TPP1 (TPP1LAMP1 mice) on the *Cln2^R207X/R207X^* genetic background, and then crossed TPP1LAMP1 mice with Vgat-Cre mice. These Vgat-Cre; TPP1LAMP1 mice accumulated storage material in cortical and striatal interneurons. Vgat-Cre; TPP1LAMP1 mice also died more readily after pentylenetetrazole-induced seizures, indicating that interneuron-specific TPP1 deficiency renders these mice more susceptible to seizure-induced mortality. We also selectively activated interneurons using designer receptors exclusively activated by designer drugs (DREADDs) in Vgat-Cre; *Cln2^R207X/R207X^* mice. Electroencephalogram monitoring revealed that DREADD-mediated activation of interneurons markedly accelerated the onset of spontaneous seizures and seizure-associated death in Vgat-Cre; *Cln2^R207X/R207X^* mice, suggesting that modulating interneuron activity can exacerbate epileptiform abnormalities. Taken together, these results provide mechanistic insights into the underlying etiology of seizures and premature death that characterize CLN2 disease.

## Abstract 2

Seizures are an important part of CLN2 disease, killing our mouse model. We show that key interneuron populations contribute mechanistically to these seizure phenotypes

## Graphical abstract

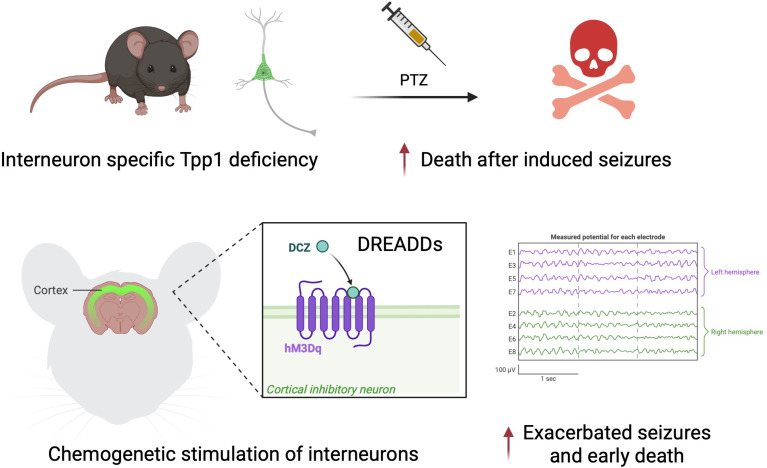

## Introduction

GABAergic interneuron deficits lead to insufficient inhibition or disinhibition, causing an imbalance between excitatory and inhibitory influences within local neural circuits (1–3). Such excitatory/inhibitory imbalance has been implicated in various neurological conditions, including epileptic seizures, and several neurodegenerative and psychiatric disorders (4–6). While selective loss or dysfunction of interneurons has been documented in numerous neurological diseases (7–12), such evidence primarily stems from descriptive studies, and direct evidence delineating a causal relationship between interneuron deficits and neurological symptoms remains limited. Filling such knowledge gaps is critical not only for elucidating the functional contribution of inhibitory networks to disease pathogenesis but also for developing potential interneuron-targeted therapeutic strategies.

The neuronal ceroid lipofuscinoses (NCLs) are a group of neurodegenerative lysosomal storage disorders (LSDs) affecting children and young adults (13). CLN2 disease, one of the most common forms, is caused by genetic defects in the lysosomal serine protease tripeptidyl peptidase 1 (TPP1), which is encoded by the *CLN2/TPP1* gene (14, 15). Clinically, children affected by CLN2 disease manifest new-onset epileptic seizures between 2 and 4 years of age, followed by ataxia, motor decline, visual loss, and premature mortality (16). Although cerliponase alfa, an FDA-approved disease-modifying enzyme replacement therapy, now exists (17), this treatment only slows but fails to halt disease progression (18, 19). Notably, managing seizures poses a substantial challenge in CLN2 disease clinical care, as seizures are often polymorphic (e.g., generalized tonic-clonic, myoclonic, atonic), progressively worsen, and become therapy resistant as the disease advances (20). Despite the urgent need for more effective treatments, little is known about the cellular mechanisms through which TPP1 deficiency triggers epilepsy and neurodegeneration (21, 22), hindering the identification of novel therapeutic targets and advancement of alternative treatment strategies for CLN2 disease.

Our previous investigation in *Cln2^R207X/R207X^*-knockin mice harboring a common disease-causing mutation (23) revealed a clinically relevant seizure phenotype as a major contributor to premature death (24). Additionally, we also revealed that *Cln2^R207X/R207X^* mice exhibit early loss of several cortical interneuron populations, including those positive for parvalbumin (PV), somatostatin (SST), calretinin (CR), and calbindin (CB) (24). While interneuron loss has also been identified in CLN2 patients (25) and a *CLN2^R208X/R208X^* porcine model (26), a key finding from our longitudinal characterization of *Cln2^R207X/R207X^* mice is that this interneuron loss precedes an onset of cortical pyramidal neuron loss and spontaneous seizures (24). This prompted us to explore a possible causal link between fatal seizures and cortical interneuron loss in *Cln2^R207X/R207X^* mice.

Studying cell-autonomous effects of TPP1 deficiency is complicated due to TPP1 being secreted from cells and taken up by neighboring cells via the mannose-6-phosphate receptor–mediated (M6PR-mediated) endocytosis pathway (27, 28). This process is known as “cross-correction,” and prevents a cell-autonomous response following cell-type-specific TPP1 deletion. To address this, we generated transgenic mice that express a lysosomal membrane–tethered version of TPP1 (TPP1LAMP1 mice) to suppress cross-correction. TPP1LAMP1 mice are on the *Cln2^R207X/R207X^* genetic background, and ubiquitous expression of the TPP1LAMP1 transgene is designed to suppress their CLN2-disease-associated phenotype. Because this transgene is flanked by *loxP* sites, the *Cre-loxP* system can be used to produce TPP1 deficiency by excising this transgene in a specific cell type. In this study, crossing TPP1LAMP1 mice with pan-interneuron *Vgat-Cre* driver mice achieved interneuron-specific TPP1 deficiency. These mice exhibited a shorter lifespan following chemically induced seizures, indicating impaired suppression of fatal seizures. Additionally, we employed a chemogenetic strategy using designer receptors exclusively activated by designer drugs (DREADDs) to selectively activate interneuron activity in *Cln2^R207X/R207X^* mice. Our results demonstrate that chronic DREADD activation of interneurons accelerates the course of spontaneous seizures and associated death in *Cln2^R207X/R207X^* mice. These findings support our hypothesis that interneurons play a mechanistic role in certain aspects of fatal seizure phenotypes in *Cln2^R207X/R207X^* mice.

## Results

### A membrane-tethered in vivo model of TPP1 enables studying the cell-autonomous effects of TPP1 deficiency.

**Figure 1:**
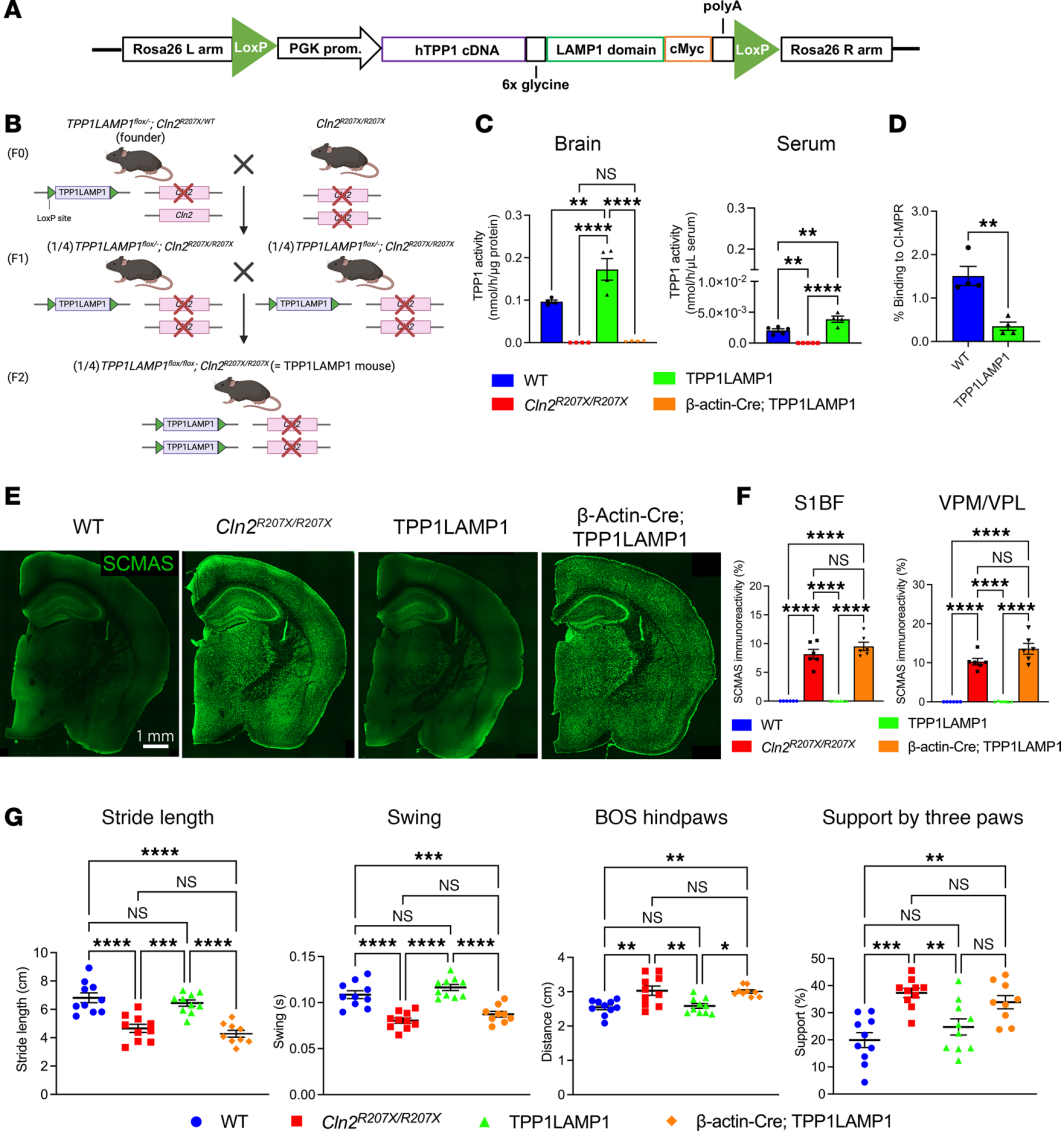
Generation of TPP1LAMP1 mice. (**A**) The TPP1LAMP1 construct was created by linking human TPP1 cDNA with the transmembrane domain of LAMP1 via a 6-glycine linker under the PGK promoter. The entire construct was flanked by *loxP* loci. (**B**) Schematic describing the breeding strategy to generate TPP1LAMP1 mice. (**C**) TPP1 activity assays in the brain (left) at 15 weeks show supraphysiological TPP1 activity in TPP1LAMP1 mice vs. WT mice, and indistinguishable TPP1 activity in β-actin-Cre; TPP1LAMP1 mice from *Cln2^R207X/R207X^* mice (*n* = 4 per group). TPP1 activity assays (right) show supraphysiological TPP1 activity in the serum of TPP1LAMP1 mice compared with WT (*n* = 5 in WT and *Cln2^R207X/R207X^* mice and *n* = 4 in TPP1LAMP1 mice). (**D**) Binding to CI-M6PR is significantly reduced in TPP1LAMP1 compared with endogenous WT TPP1 in the brain (*n* = 4 per group). (**E**) Immunostaining for SCMAS (green) shows widespread SCMAS accumulation in *Cln2^R207X/R207X^* mice and β-actin-Cre; TPP1LAMP1 mice, but not in WT and TPP1LAMP1 mice at 15 weeks. Scale bar: 1 mm. (**F**) Quantitative analysis of SCMAS immunoreactivity in the S1BF and VPM/VPL at 15 weeks confirms that SCMAS accumulation seen in *Cln2^R207X/R207X^* mice (red bars) is completely rescued in TPP1LAMP1 mice (green bars) and is fully recapitulated in β-actin-Cre; TPP1LAMP1 mice (purple bars) across multiple brain regions (*n* = 6 per group). (**G**) CatWalk XT gait analysis shows significantly shorter stride length and swing duration, wider distance between hind paws (base of support or BOS), and higher proportion of steps supported by 3 feet in 15-week-old *Cln2^R207X/R207X^* mice (red) compared with age-matched WT mice (blue). These gait abnormalities were rescued in TPP1LAMP1 mice (green) and recapitulated in β-actin-Cre; TPP1LAMP1 mice (purple) at the same age (*n* = 10 in WT, *Cln2^R207X/R207X^*, and TPP1LAMP1 mice and *n* = 9 in β-actin-Cre; TPP1LAMP1). Dots represent values from individual animals. Values are shown as mean ± SEM. One-way ANOVA with Bonferroni’s correction (**C**, **F**, and **G**) and unpaired, 2-tailed *t* test (**D**). *P < 0.05; **P < 0.01; ***P < 0.001; ****P < 0.0001.

Investigating the contribution of interneuron deficits to CLN2 disease pathogenesis required the generation of a new conditional *Tpp1*-mutant mouse. To prevent cross-correction of neighboring TPP1-deficient cell types by secreted enzyme, we adopted a membrane-tethering approach that was previously validated in a mouse model of Krabbe disease, which is caused by a deficiency of a different lysosomal enzyme, galactocerebrosidase (GALC) (29). We designed a chimeric fusion protein in which human TPP1 is tethered to the lysosomal membrane by linking TPP1 to the transmembrane domain and cytosolic tail of the lysosomal associated membrane protein 1 (LAMP1) via a 6-glycine linker (Figure 1A). Lentivirus-driven overexpression of the chimeric TPP1LAMP1 in mouse embryonic fibroblasts (MEFs) derived from a *Cln2*-null (*Cln2*^–/–^) mutant mouse (30) rescued intracellular TPP1 enzymatic activity but did not result in extracellular secretion of TPP1 into the conditioned media or cross-correction in cocultured *Cln2*^–/–^ MEFs (Supplemental Figure 1, A and B; supplemental material available online with this article; https://doi.org/10.1172/jci.insight.184487DS1).

Having validated the function of TPP1LAMP1 protein in vitro, we generated a transgenic mouse that ubiquitously expresses *loxP*-flanked TPP1LAMP1 under a PGK promotor on the *Cln2^R207X/R207X^* genetic background using a CRISPR/Cas9-mediated targeting strategy. The design of this ubiquitous expression of the *loxP*-flanked TPP1LAMP1 transgene was intended to normalize disease phenotypes of *Cln2^R207X/R207X^* mice and enable conditional elimination of TPP1 enzymatic activity using the *Cre-loxP* system. After confirming the germline insertion of the TPP1LAMP1 transgene into the *Rosa26* locus in founder mice (Supplemental Figure 2A), we crossed the founder mice with *Cln2^R207X/R207X^* to achieve double homozygosity for *TPP1LAMP1^flox^* and *Cln2^R207X^* (*TPP1LAMP1^fl/fl^;* *Cln2^R207X/R207X^* mice, henceforth referred to as TPP1LAMP1 mice) (Figure 1B). PCR analysis on mRNA-derived complementary DNA (cDNA) from the liver tissue of TPP1LAMP1 mice confirmed that TPP1LAMP1 is transcribed as a single unit (Supplemental Figure 2B). TPP1 activity assays in total brain protein extracts also confirmed supraphysiological TPP1 enzymatic activity in TPP1LAMP1 mice in vivo compared with WT mice (Figure 1C). However, TPP1 assays in the serum unexpectedly revealed very low, but detectable, extracellular TPP1 enzymatic activity both in TPP1LAMP1 and WT mice (Figure 1C), in contrast with our aforementioned in vitro findings (Supplemental Figure 1, A and B). Because this unanticipated low level of circulating TPP1 could undermine the usefulness of the TPP1LAMP1 mouse as a tool for testing cellular autonomy, we investigated whether this enzyme could bind to the cation-independent M6PR (CI-M6PR) on the surface of neighboring cells and be delivered into lysosomes by performing a CI-M6PR affinity assay on the total brain protein extracts from TPP1LAMP1 and WT mice. The analysis revealed markedly reduced (~77%) affinity of TPP1LAMP1 for the CI-M6PR beads compared with endogenous TPP1 (Figure 1D), in line with previous in vitro reports (29). Taken together, these data from in vivo samples demonstrate that although membrane tethering does not completely prevent extracellular secretion of TPP1, it substantially restricts cross-correction of the enzyme, validating TPP1LAMP1 mice as an appropriate model to study the cell-autonomous function of TPP1 deficiency.

Next, we verified whether expression of TPP1LAMP1 rescues CLN2-disease-associated phenotypes at 15 weeks (3.5 months) of age, which represents disease end stage for *Cln2^R207X/R207X^* mice. This is an important prerequisite for demonstrating that the enzyme expressed by our TPP1LAMP1 transgene is biologically active in vivo. Storage material accumulation is the characteristic pathological hallmark of LSDs (31, 32), and *Cln2^R207X/R207X^* mice display pronounced accumulation of subunit C of mitochondrial ATPase (SCMAS) throughout the central nervous system (24). Immunohistochemistry revealed that SCMAS accumulation was fully prevented in the brain of TPP1LAMP1 mice (Figure 1E), which was further confirmed by quantitative thresholding image analysis within somatosensory pathways, including the primary somatosensory barrel cortex (S1BF) and the ventral posterior medial/lateral thalamic nuclei (VPM/VPL), which are the most affected brain regions in *Cln2^R207X/R207X^* mice (Figure 1F). Gait abnormalities are also observed in *Cln2^R207X/R207X^* mice at disease end stage (24), and CatWalk XT gait analysis showed fully preserved gait performance in TPP1LAMP1 mice (Figure 1G). These results demonstrate that TPP1LAMP1 mice are histologically and behaviorally as healthy as WT mice.

As a final step of validation, we tested whether *Cre-loxP* recombination efficiently excises the *loxP*-flanked TPP1LAMP1 transgene, by crossing TPP1LAMP1 mice with *Actb-Cre* mice, which ubiquitously express *Cre* (*Actb-Cre^+/–^; TPP1LAMP1^fl/–^;* *Cln2^R207X/R207X^* mice, henceforth referred to as β-actin-Cre; TPP1LAMP1 mice). PCR analysis targeting the entire TPP1LAMP1 insert at the *Rosa26* locus revealed the emergence of a new amplicon that is consistent with the length of the floxed allele (Supplemental Figure 2C), indicating that the *loxP*-flanked TPP1LAMP1 allele was properly excised. In agreement with this result, TPP1 activity assay in total brain protein extracts revealed dramatically reduced enzymatic activity (2.8% of WT) in β-actin-Cre; TPP1LAMP1 mice, which was comparable to TPP1 activity levels in *Cln2^R207X/R207X^* mice (Figure 1C). Histological analysis and CatWalk XT gait analysis showed widespread SCMAS accumulation and gait abnormalities, respectively, in β-actin-Cre; TPP1LAMP1 mice to a similar extent as in *Cln2^R207X/R207X^* mice (Figure 1, E–G). Collectively, these results confirm that ubiquitous *Cre-loxP*–mediated TPP1LAMP1 excision efficiently recapitulates disease phenotypes of *Cln2^R207X/R207X^* mice, validating TPP1LAMP1 mice as an ideal platform to study cell-autonomous effects of conditional TPP1 deficiency in vivo.

### Interneuron-specific TPP1 deficiency partially recapitulates neuropathology of CLN2 disease.

To elicit TPP1 deficiency in multiple GABAergic interneuron populations, we selected *Vgat-Cre* mice in which *Cre* was inserted downstream of the solute carrier family 32 (GABA vesicular transporter), member 1 gene (*Slc32a1* or *Vgat*) (33). To validate that *Cre* expression was present within these interneuron populations in *Vgat-Cre* mice, we first crossed *Vgat-Cre* mice with *Ai14* mice, a reporter strain that expresses tdTomato in the presence of *Cre* (34). Histological analysis of the brain sections from *Vgat-Cre^+/–^; Ai14^+/–^* mice revealed that major inhibitory neuron populations, including PV-, SST-, and CR-positive interneurons within the S1BF, colocalized with tdTomato (Supplemental Figure 3, A and B). In the S1BF, 32.9%, 26.1%, and 8.3% of tdTomato-positive cells were PV-, SST-, and CR-positive neurons, respectively (Supplemental Figure 3C), and this composition is largely consistent with the previous literature (35, 36). We also found that COUP-TF–interacting protein 2–positive (CTIP2-positive) interneurons within the caudate putamen (CPu) in the striatum, known as medium-sized spiny neurons (MSNs), also colocalized with the tdTomato signal (Supplemental Figure 3, A and B). Taken together, these results confirm that *Vgat-Cre* effectively targets interneurons in multiple brain regions.

**Figure 2:**
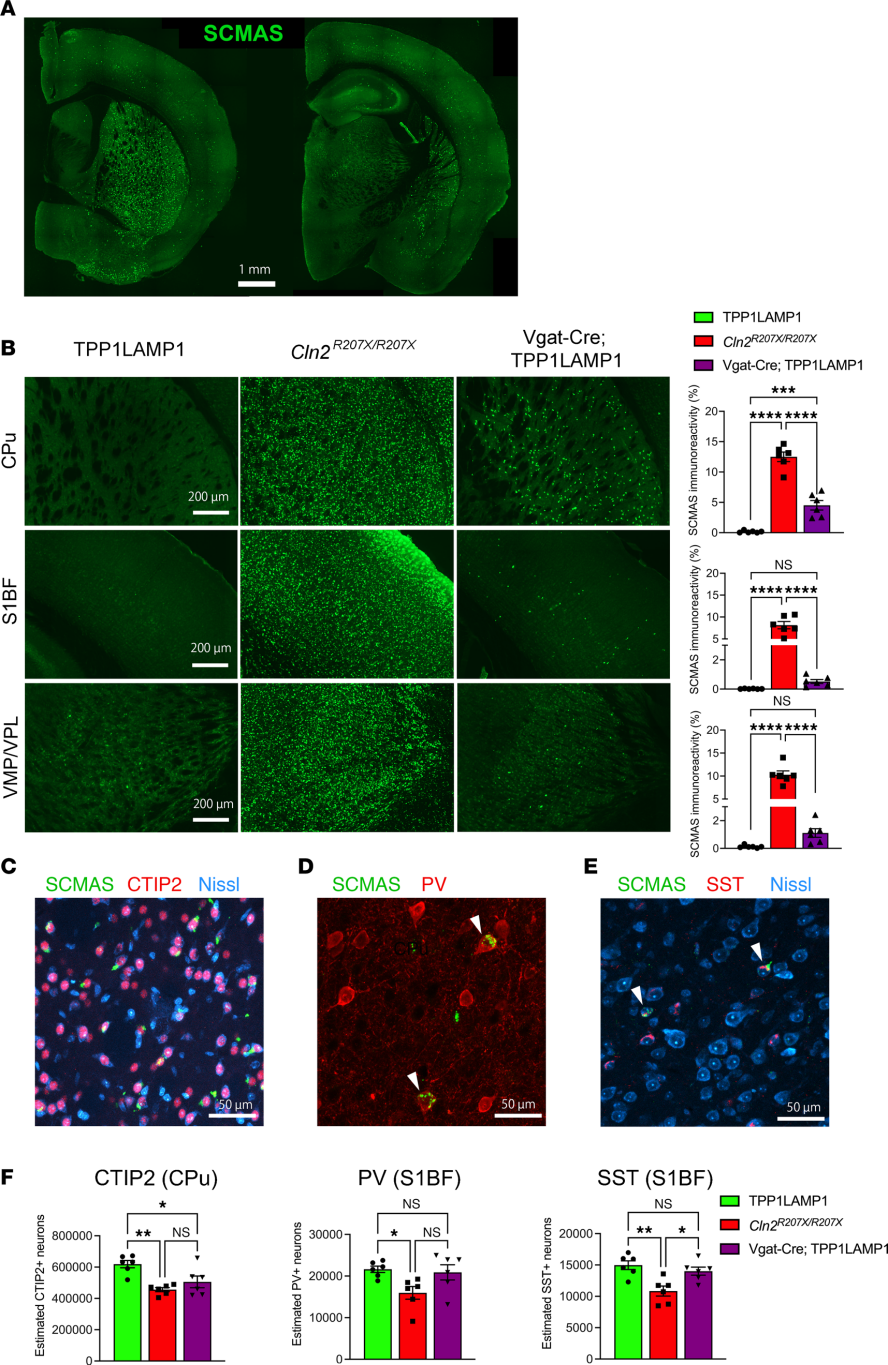
Interneuron-specific TPP1 deficiency leads to storage material accumulation and interneuron loss. (**A**) Immunostaining for SCMAS (green) in the brain of Vgat-Cre; TPP1LAMP1 at 15 weeks of age shows scattered distribution of SCMAS-positive cells across the brain. (**B**) Comparison of immunostained brain sections for SCMAS (green) and quantitative analysis of their immunoreactivity via thresholding image analysis between TPP1LAMP1, *Cln2^R207X/R207X^*, and Vgat-Cre; TPP1LAMP1 mice across multiple brain regions reveals partial storage material accumulation in Vgat-Cre; TPP1LAMP1 compared with *Cln2^R207X/R207X^* mice, with statistically significant differences observed within the CPu at 15 weeks of age. (**C**) Coimmunostaining for SCMAS (green), CTIP2 (red), and Nissl (cyan) reveals storage material accumulation in a subset of CTIP2-positive MSNs within the CPu in Vgat-Cre; TPP1LAMP1 mice. (**D**) Coimmunostaining for SCMAS (green) and PV (red) reveals storage material accumulation in a subset of PV-positive interneurons within the S1BF in Vgat-Cre; TPP1LAMP1 mice. (**E**) Coimmunostaining for SCMAS (green), SST (red), and Nissl (cyan) reveals storage material accumulation in a subset of SST-positive interneurons within the S1BF cortex in Vgat-Cre; TPP1LAMP1 mice. (**F**) Unbiased stereological counts of immunostained neuron populations within the CPu reveals a significant loss of CTIP2-positive medium spiny neurons in Vgat-Cre; TPP1LAMP1 mice to a comparable extent to that in *Cln2^R207X/R207X^* mice at 15 weeks of age. The same unbiased stereological analysis within the S1BF cortex reveals no significant loss of PV- or SST-positive neurons in Vgat-Cre; TPP1LAMP1 mice compared to those in TPP1LAMP1 mice at 15 weeks of age. Dots represent values from individual animals. Values are shown as mean ± SEM (*n* = 6 mice per group). One-way ANOVA with Bonferroni’s correction. *P < 0.05; **P < 0.01; ***P < 0.001; ****P < 0.0001. Scale bars: 1 mm (**A**), 200 μm (**B**), and 50 μm (**C**–**E**).

Next, we crossed TPP1LAMP1 mice with *Vgat-Cre* mice to introduce interneuron-specific TPP1 deficiency (*Vgat-Cre^+/–^; TPP1LAMP1^fl/fl^;* *Cln2^R207X/R207X^* mice, henceforth referred to as Vgat-Cre; TPP1LAMP1 mice). Immunostaining for SCMAS, the key pathological hallmark of CLN2 disease, in the brain of Vgat-Cre; TPP1LAMP1 mice at 15 weeks of age showed scattered distribution of SCMAS-positive cells across the brain (Figure 2, A and B). Threshold image analysis revealed significantly increased SCMAS immunoreactivity within the CPu of Vgat; TPP1LAMP1 compared with TPP1LAMP1 mice (Figure 2B). To confirm whether these SCMAS-positive cells are interneurons, we counterstained brain sections of Vgat-Cre; TPP1LAMP1 mice for CTIP2, PV, and SST. Coimmunostaining revealed that SCMAS accumulated in a subset of CTIP2-positive MSNs within the CPu and PV- and SST-positive interneurons within the S1BF (Figure 2, C–E), suggesting successful TPP1 deletion in these interneuron populations. To determine whether interneuron survival was affected in Vgat-Cre; TPP1LAMP1 mice, we performed unbiased stereological counting on immunostained brain sections. Statistical analysis revealed a significant loss of CTIP2-positive MSNs within the CPu in Vgat-Cre; TPP1LAMP1 mice compared with age-matched TPP1LAMP1 mice, which was indistinguishable from age-matched *Cln2^R207X/R207X^* mice (Figure 2F). However, the same unbiased stereological analysis within the S1BF revealed no significant reduction in PV- or SST-positive neurons in Vgat-Cre; TPP1LAMP1 mice compared to age-matched TPP1LAMP1 mice (Figure 2F), suggesting a subpopulation-dependent impact of TPP1 deficiency on interneuron loss in Vgat-Cre; TPP1LAMP1 mice.

**Figure 3:**
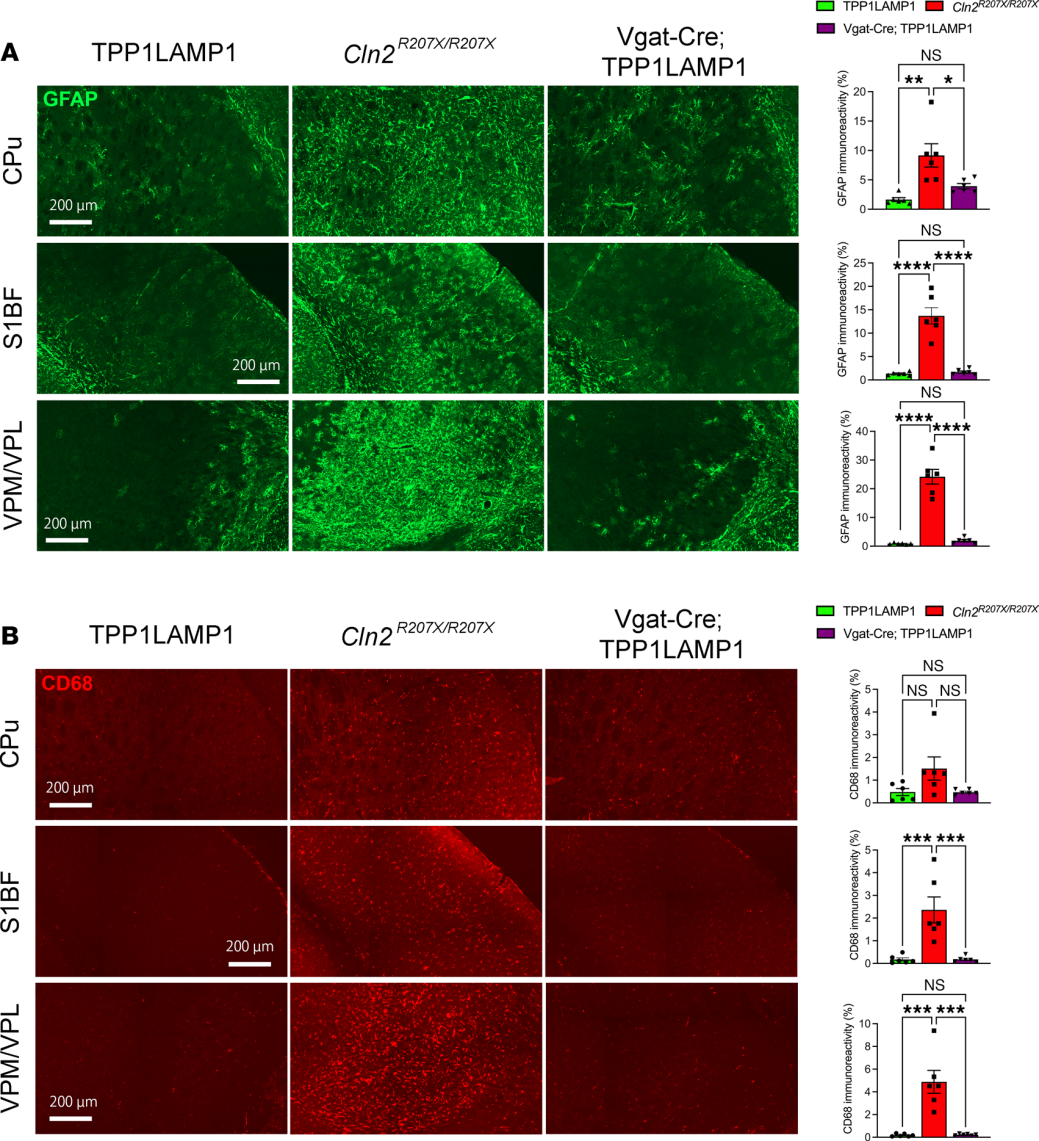
Interneuron-specific TPP1 deficiency does not trigger the neuroimmune response associated with CLN2 disease. Immunostaining for GFAP (**A**, green) and CD68 (**B***,* red) and quantitative analysis of their immunoreactivity via thresholding image analysis in the CPu, S1BF, and VPM/VPL at 15 weeks of age shows the marked increase in the intensity of GFAP and CD68 immunoreactivities in *Cln2^R207X/R207X^* mice (red bars), but no significant increase in Vgat-Cre; TPP1LAMP1 mice (purple bars) compared to age-matched TPP1LAMP1 mice (green bars). Dots represent values from individual animals. Values are shown as mean ± SEM (*n* = 6 mice per group). One-way ANOVA with Bonferroni’s correction. *P < 0.05; **P < 0.01; ***P < 0.001; ****P < 0.0001. Scale bars: 200 μm.

Localized glial activation of both astrocytes and microglia is another pathological hallmark of NCLs, including CLN2 disease (37). To assess whether interneuron-specific TPP1 deficiency triggers glial activation, we immunostained brain sections from 15-week-old Vgat-Cre; TPP1LAMP1 mice for glial fibrillary acidic protein (GFAP), a marker for astrogliosis, and CD68, a marker for microglial activation (22, 37). This analysis revealed a marked increase in both GFAP and CD68 immunoreactivities in age-matched *Cln2^R207X/R207X^* mice across the CPu, S1BF, and VPM/VPL, but no significant increase was observed within any of these brain regions of Vgat-Cre; TPP1LAMP1 mice compared to age-matched TPP1LAMP1 mice (Figure 3, A and B). These results suggest that TPP1-deficient interneurons do not trigger a non–cell-autonomous neuroimmune response associated with CLN2 disease.

### Interneuron-specific TPP1 deficiency increases susceptibility to sudden death secondary to PTZ-induced seizures.

**Figure 4:**
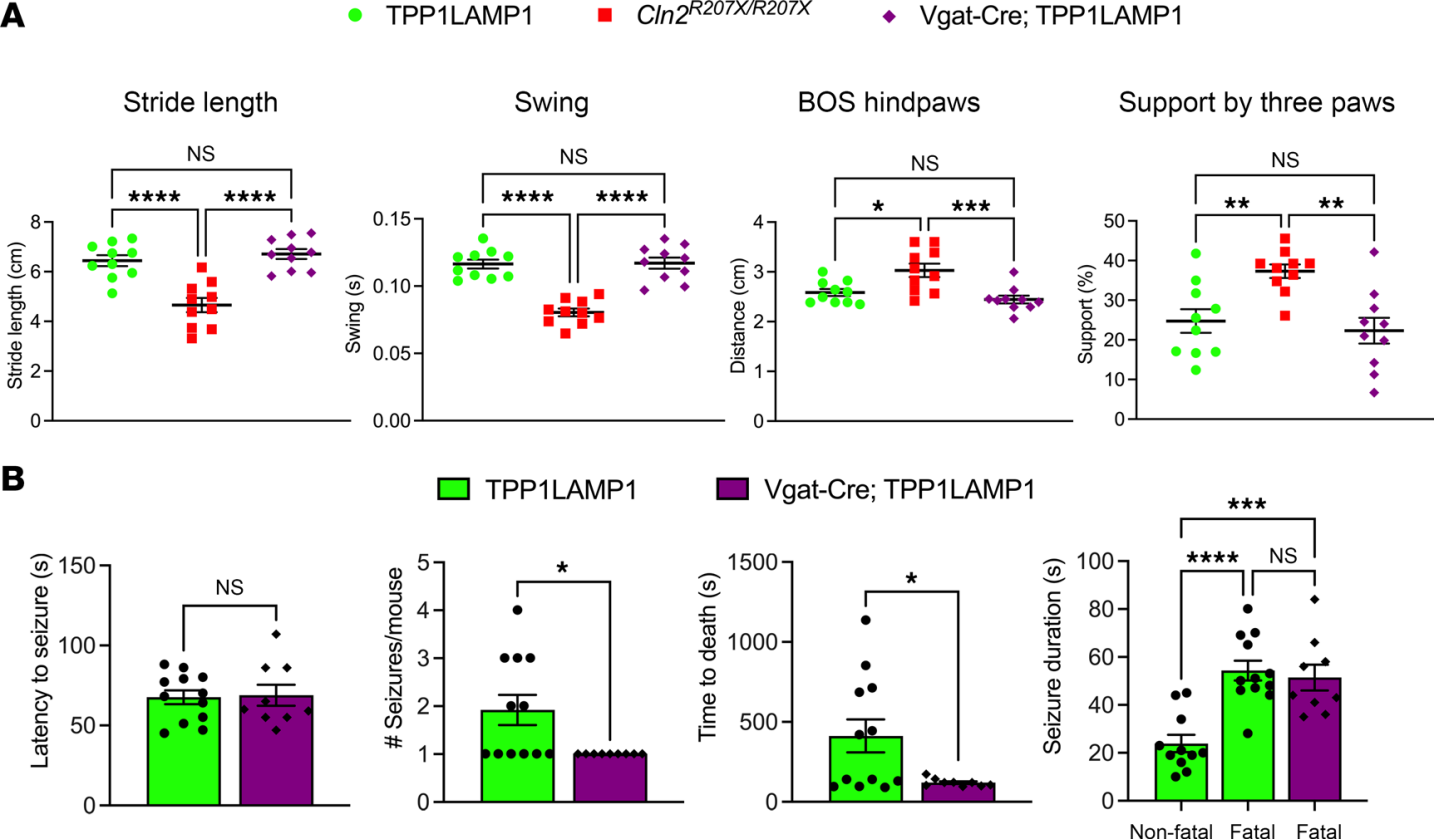
Interneuron-specific TPP1 deficiency increases susceptibility to sudden death secondary to pentylenetetrazole-induced seizures. (**A**) CatWalk XT gait analysis reveals preserved gait performance of Vgat-Cre; TPP1LAMP1 mice (purple) compared with TPP1LAMP1 and *Cln2^R207X/R207X^* mice at 15 weeks of age (*n* = 10 per group). (**B**) Seizures were induced by intraperitoneal injection of pentylenetetrazole (PTZ; 75 mg/kg) in TPP1LAMP1 (*n* = 12, green bars) and Vgat-Cre; TPP1LAMP1 (*n* = 9, purple bars) mice at 25 weeks of age. There was no significant difference in the latency to tonic-clonic seizures between 2 groups. Vgat-Cre; TPP1LAMP1 mice exhibit a significantly lower number of tonic-clonic seizures per mouse and a significantly shorter time to death compared with TPP1LAMP1 mice. The average duration of nonfatal tonic-clonic seizures in TPP1LAMP1 mice was significantly shorter than the average duration of fatal tonic-clonic seizures in TPP1LAMP1 mice and Vgat-Cre; TPP1LAMP1 mice. Dots represents values from individual animals. Values are shown as mean ± SEM. One-way ANOVA with Bonferroni’s correction (**A**) and unpaired, 2-tailed *t* test (**B**). *P < 0.05; **P < 0.01; ***P < 0.001; ****P < 0.0001.

We then investigated whether Vgat-Cre; TPP1LAMP1 mice demonstrate any neurological phenotypes, including the gait disturbance and seizure phenotypes associated with CLN2 disease. CatWalk XT gait analysis revealed no significant deterioration of gait parameters in 15-week-old Vgat-Cre; TPP1LAMP1 mice compared to age-matched TPP1LAMP1 mice (Figure 4A). The same gait analysis was repeated at an older age (25 weeks old), yet no significant change was observed between Vgat-Cre; TPP1LAMP1 and TPP1LAMP1 mice (data not shown). These data suggest no apparent impact of interneuron-specific TPP1 deficiency on gait performance.

Next, we performed long-term EEG monitoring on Vgat-Cre; TPP1LAMP1 mice. We previously reported that *Cln2^R207X/R207X^* mice start to display abnormal background EEG activity such as epileptiform spikes and spike bursts from approximately 12 weeks of age onwards and subsequently develop spontaneous tonic-clonic seizures with a median onset of 15 weeks of age (24). However, EEG monitoring in Vgat-Cre; TPP1LAMP1 mice revealed no apparent interictal epileptiform background abnormalities or spontaneous seizures up to 30 weeks of age. Therefore, we induced seizures in both TPP1LAMP1 (*n* = 12) and Vgat-Cre; TPP1LAMP1 mice (*n* = 9) at 25 weeks of age by intraperitoneal injection of pentylenetetrazole (PTZ) at 75 mg/kg body weight. This approach provided a more sensitive measure to interrogate whether interneuron-specific TPP1 deficiency affects seizure susceptibility. Our analysis revealed no significant difference in the average latency to tonic-clonic seizures (Figure 4B), suggesting that the threshold for seizure initiation is not altered in Vgat-Cre; TPP1LAMP1 mice. However, while half (6 of 12) of the TPP1LAMP1 mice died after a single seizure and the remaining mice died after experiencing multiple seizures, in contrast all (9 of 9) of the Vgat-Cre; TPP1LAMP1 mice died immediately after a single tonic-clonic seizure (*P* = 0.0186, Fisher’s exact test). This resulted in a significantly lower average number of tonic-clonic seizures before dying and a significantly shorter average time of death in Vgat-Cre; TPP1LAMP1 mice compared with TPP1LAMP1 mice (Figure 4B). Furthermore, we found that the average duration of nonfatal tonic-clonic seizures in TPP1LAMP1 mice was significantly lower than the average duration of fatal tonic-clonic seizures in TPP1LAMP1 mice and Vgat-Cre; TPP1LAMP1 mice. Taken together, these results suggest that Vgat-Cre; TPP1LAMP1 mice are less capable of terminating PTZ-induced seizures, and thus more prone to die following these pharmacologically induced seizures compared with TPP1LAMP1 mice.

### Chemogenetic activation of interneurons exacerbates seizure phenotypes in Cln2^R207X/R207X^ mice.

**Figure 5:**
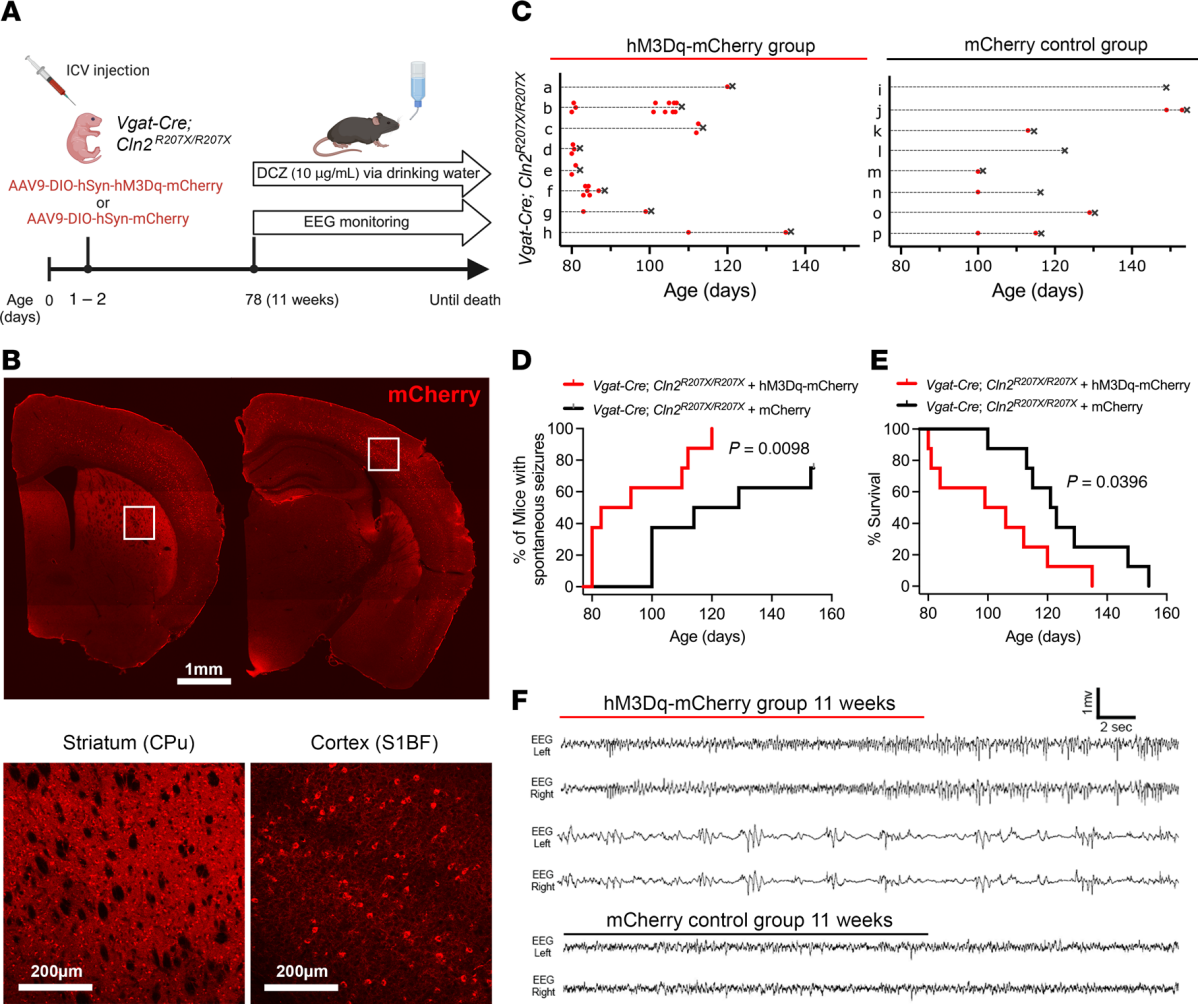
Chemogenetic activation of interneurons exacerbates seizure phenotypes in *Cln2^R207X/R207X^* mice. (**A**) Schematic of experimental design. The image was created with BioRender.com. (**B**) Fluorescence images depict widespread transduction of intracerebroventricularly delivered AAV9-hSyn-DIO-hM3Dq-mCherry (red) in *Vgat-Cre*; *Cln2^R207X/R207X^* mouse brain at 6 weeks of age, predominantly in the cortex and striatum. Lower-magnification images (top) and higher-magnification images focused on the CPu and S1BF (bottom). Scale bars: 1 mm (top) and 200 μm (bottom). (**C**) Time course of seizures (red dots) and deaths (black Xs) in hM3Dq-mCherry–expressing (top) and mCherry-expressing (bottom) *Vgat-Cre*; *Cln2^R207X/R207X^* mice. EEG recordings revealed a significantly earlier onset of spontaneous seizures (**D**) and premature death (**E**) in hM3Dq-mCherry–expressing *Vgat-Cre*; *Cln2^R207X/R207X^* mice upon chronic deschloroclozapine (DCZ) administration compared with mCherry-expressing control mice (*n* = 8 per group). Log-rank (Mantel-Cox) test. (**F**) Representative EEG traces show an increased frequency of abnormal spikes (top) and burst-suppression activity (middle) in hM3Dq-mCherry–expressing *Vgat-Cre*; *Cln2^R207X/R207X^* mice during the first week of DCZ administration compared with mCherry-expressing *Vgat-Cre*; *Cln2^R207X/R207X^* mice (bottom).

As a complementary approach to investigate the contribution of interneurons to seizure phenotypes in *Cln2^R207X/R207X^* mice, we chemogenetically activated interneurons using hM3Dq, a modified form of the human M3 muscarinic (hM3) receptor classified as a DREADD. *Cre*-dependent AAV9-hSyn-DIO-hM3Dq-mCherry or AAV9-hSyn-DIO-mCherry (positive control, *n* = 8 per group) was intracerebroventricularly injected into neonatal (P1–P2) *Vgat-Cre^+/–^;* *Cln2^R207X/R207X^* mice to transduce hM3Dq-DREADD in interneurons across the brain, predominantly in the cortex and striatum (Figure 5, A and B). At 78 days (11 weeks, 0 days) of age, which is just before *Cln2^R207X/R207X^* mice start to display epileptiform abnormalities, we started to chronically administer 10 μg/mL deschloroclozapine (DCZ), a recently described DREADD ligand that appears to be more potent and specific compared with other classic DREADD ligands (38, 39), via drinking water in both treatment groups (Figure 5A). While DREADD ligands have been more commonly administered by intraperitoneal injection, this noninvasive oral administration route of DCZ was selected to minimize any additional handling stress that could potentially induce seizures in *Cln2^R207X/R207X^* mice. Long-term EEG monitoring was also initiated at 78 days of age and continued until the mice died. The hM3Dq-DREADD–activated group showed a significantly earlier onset of spontaneous seizures along with earlier onset of interictal abnormalities, including epileptiform spikes and burst-suppression pattern, compared with the mCherry-expressing control group (Figure 5, C, D, and F). This led to significantly earlier death in the hM3Dq-DREADD–activated group compared with the mCherry control group (Figure 5, C and E). As a negative control study, we also chemogenetically activated interneurons in healthy WT mice (*n* = 6) by injecting *Cre*-dependent AAV9-hSyn-DIO-hM3Dq-mCherry into *Vgat-Cre* mice on the WT background for the *Cln2* allele. Unexpectedly, EEG monitoring showed early-onset seizures in the majority of the hM3Dq-DREADD–activated WT mice, along with premature death in half of the cohort by 22 weeks of age (Supplemental Figure 4, A–C). These findings suggest that chronic activation of interneurons alone could induce seizures and contribute to fatality, albeit to a much lesser extent than in hM3Dq-DREADD–activated *Cln2^R207X/R207X^* mice, all of which died immediately following seizures. Taken together, these results indicate that chemogenetic chronic activation of interneurons exacerbates epileptic seizures and accelerates seizure-related death in *Cln2^R207X/R207X^* mice, providing additional evidence in support of the involvement of interneurons in epileptogenesis and premature death associated with CLN2 disease.

### Chemogenetic activation of interneurons alters microglial activation and astrocytic GABA levels in Cln2^R207X/R207X^ mice.

**Figure 6:**
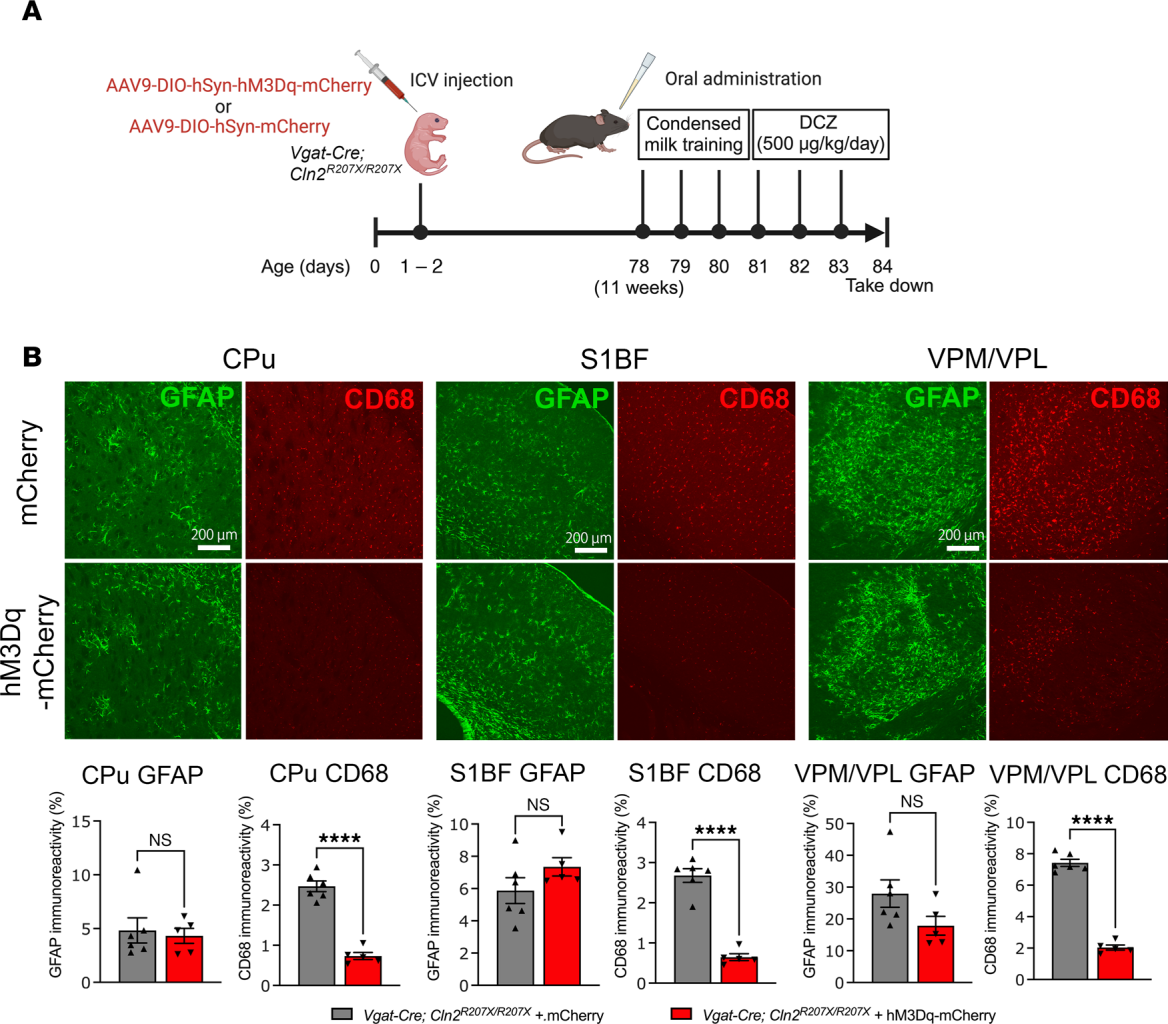
Chemogenetic activation of interneurons alters microglial activation in *Cln2^R207X/R207X^* mice. (**A**) Schematic of experimental design. The image was created with BioRender.com. (**B**) Immunostaining for GFAP (green) and CD68 (red) and quantitative analysis of their immunoreactivity via thresholding image analysis reveals a significantly reduced CD68 immunoreactivity in hM3Dq-mCherry–expressing *Vgat-Cre*; *Cln2^R207X/R207X^* mice (*n* = 5, red bars) upon DCZ administration across the CPu, S1BF, and VPM/VPL with no change in GFAP immunoreactivity compared to mCherry-expressing control mice (*n* = 6, gray bars). Scale bars: 200 μm. Dots represents values from individual animals. Values are shown as mean ± SEM. Unpaired, 2-tailed *t* test. ****P < 0.0001.

Observing how chemogenetic activation of interneurons alters seizure phenotypes in *Cln2^R207X/R207X^* mice raised the question of whether interneuron activity also affects the disease-associated neuropathological phenotypes of *Cln2^R207X/R207X^* mice. Thus, we extended our analysis to investigate the neuroimmune responses at a histological level in DREADD-treated *Vgat-Cre^+/–^;* *Cln2^R207X/R207X^* mice. For this purpose, we treated *Vgat-Cre^+/–^;* *Cln2^R207X/R207X^* mice with DCZ for 72 hours at 11 weeks of age (Figure 6A). To achieve a tighter control of daily DCZ consumption, we utilized the micropipette-guided administration method (40) rather than administration via drinking water to precisely administer 500 μg/kg/day of DCZ in condensed milk to *Vgat-Cre^+/–^;* *Cln2^R207X/R207X^* mice (Figure 6A). Immunohistological analysis revealed a significant reduction in CD68 immunoreactivity in the hM3Dq-DREADD–activated group across the CPu, S1BF, and VPM/VPL compared with the mCherry control group, whereas no significant change in GFAP immunoreactivity was observed between any two groups (Figure 6B).

**Figure 7:**
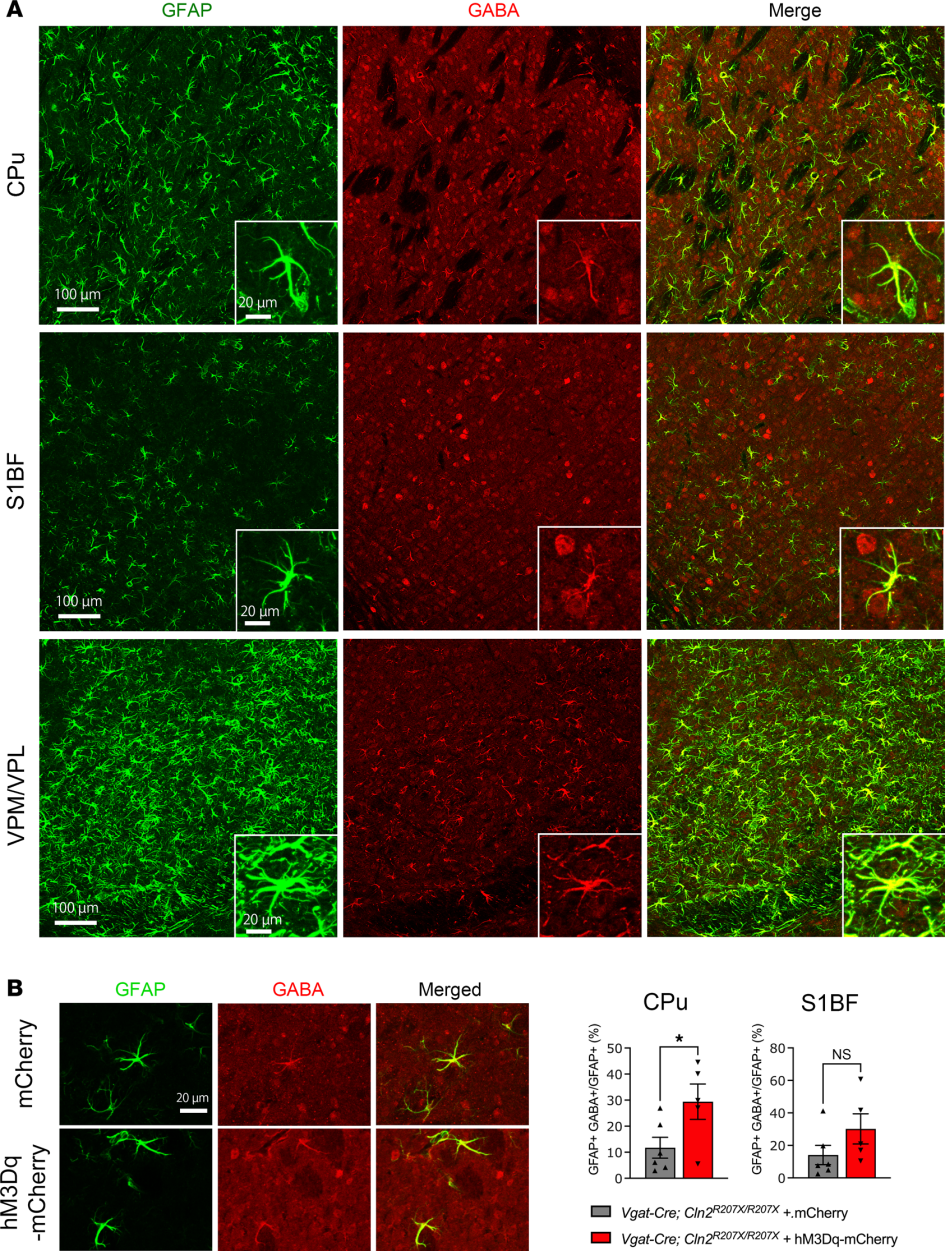
Chemogenetic activation of interneurons aggravates GABA immunoreactivity within astrocytes in *Cln2^R207X/R207X^* mice. Coimmunostaining for GFAP (green) and GABA (red) shows overlap between the 2 channels across the CPu, S1BF, and VPM/VPL in 12-week-old *Cln2^R207X/R207X^* mice. Insets are higher-magnification views from each image. Scale bars: 100 μm (low magnification) and 20 μm (high magnification). (**B**) Immunostaining for GFAP (green) and GABA (red) and colocalization analysis on the confocal images reveals a significantly increased GABA immunoreactivity in GFAP-positive astrocytes within the CPu of hM3Dq-mCherry–expressing *Vgat-Cre*; *Cln2^R207X/R207X^* mice (*n* = 5, red bars) upon DCZ administration compared with mCherry-expressing control mice (*n* = 6, gray bars). There was a similar increase in the GABA immunoreactivity in GFAP-positive astrocytes within the S1BF, but it was not statistically significant. Scale bar: 20 μm. Dots represents values from individual animals. Values are shown as mean ± SEM. Unpaired, 2-tailed *t* test. *P < 0.05.

While chemogenetic manipulation of interneuron activity appeared to have no effect on astrogliosis, considering the recent evidence suggesting astrocytic regulation of GABA (41, 42), we next explored whether astrocytic GABA expression is altered in the brain of *Cln2^R207X/R207X^* mice and whether that can be affected by chemogenetic manipulation of interneuron activity. Coimmunostaining for GFAP and GABA revealed pronounced colocalization of these 2 signals within the CPu, S1BF, and VPM/VPL as early as 12 weeks (3 months) of age (Figure 7A), suggesting increased levels of GABA in GFAP-positive astrocytes. We then performed GFAP/GABA coimmunostaining on brain sections of hM3Dq-mCherry– or mCherry-expressing *Vgat-Cre^+/–^;* *Cln2^R207X/R207X^* mice treated with DCZ for 72 hours, focusing on the CPu and S1BF as regions where AAV9-mediated hM3Dq transduction primarily occurred. Colocalization analysis of confocal images revealed a significant increase in the colocalization of GFAP and GABA within GFAP-positive structures in the CPu and a trend toward an increase within the S1BF, although it was not statistically significant (Figure 7B). Taken together, these data indicate that chemogenetic enhancement of interneuron activity increases local astrocytic GABA levels in *Cln2^R207X/R207X^* mice, suggesting a novel interaction between interneurons and astrocytes in the context of CLN2 disease.

## Discussion

The primary focus of this study was to explore whether there is any relationship between interneurons and seizure etiology in CLN2 disease mice. Data from our interneuron-specific TPP1-deficient mice, substantiated by chemogenetic manipulation of interneurons, suggest that these cell populations do indeed contribute to seizure phenotypes in *Cln2^R207X/R207X^* mice. In addition, our data reveal insights into the effects of TPP1 deficiency on striatal interneurons, and roles for both astrocytes and microglia in the pathogenesis of this fatal disorder. Importantly, we have also generated what we believe is a novel transgenic model to enable the study of cellular autonomy of TPP1 deficiency in any chosen cell type.

In this study, we have generated and demonstrated the utility of TPP1LAMP1 mice in which cross-correction is prevented as a valuable tool for investigating the cell-autonomous effects of conditional TPP1 deficiency. Cross-correction of lysosomal enzymes between cells involves both secretion into the extracellular space and subsequent endocytosis via the M6PR pathway (43). Based on our in vitro data, we initially assumed that membrane tethering would completely prevent TPP1 from being secreted extracellularly, and the unexpected detection of a low level of TPP1 enzymatic activity in the serum of TPP1LAMP1 mice prompts speculation regarding its mechanism. Since a substantial pool of LAMP1 traffics via the plasma membrane en route to the lysosome (44), it is likely that TPP1LAMP1 follows the same trafficking route in cells. Considering that TPP1 has both exopeptidase and endopeptidase functions (45–48), it is possible that TPP1LAMP1, which is normally confined to the lysosomal lumen, undergoes some autocatalytic cleavage either inside the lysosome or at the cell surface, releasing free TPP1 into the extracellular space. Alternatively, 5%–20% of newly synthesized lysosomal enzymes escape binding to M6PR and are secreted (49), and it is possible that TPP1LAMP1 might be prone to extracellular secretion. Another possibility is that TPP1LAMP1 might exist inside exosomes or extracellular vesicles released into the extracellular space, and evidence suggests that LAMP1 is present in neuron-derived plasma exosomes (50). Nevertheless, both TPP1LAMP1 and previously studied GALCLAMP1 (29) exhibit markedly reduced affinity for CI-M6PR compared with native enzymes. This suggests that membrane tethering of lysosomal enzymes markedly alters their mannose-6 phosphorylation, thus evading recognition by, and binding to, the cell-surface-expressed M6PR, preventing their usual ability to cross-correct neighboring cells. Our histological data provide further support for the idea that TPP1 cross-correction is effectively prevented in TPP1LAMP1 mice. Staining for SCMAS demonstrated accumulation of this sensitive marker of TPP1 deficiency within interneurons in Vgat-Cre; TPP1LAMP1 mice, despite being surrounded by phenotypically healthy cell types. Therefore, despite the unexpected presence of a low level of extracellular TPP1 activity in serum, TPP1LAMP1 mice remain a valid model for assessing conditional TPP1 deficiency.

We included the CPu, the largest area of the murine striatum, in our histological analysis since pronounced SCMAS accumulation was evident in many striatal cells in Vgat-Cre; TPP1LAMP1 mice. GABAergic MSNs account for approximately 95% of striatal neurons in rodents (51), receiving glutamatergic inputs from the cortex and thalamus, and dopaminergic inputs from the substantia nigra pars compacta (52). Loss or dysfunction of these striatal MSNs has been implicated in movement disorders such as Parkinson and Huntington diseases (53, 54). Although neuron loss and glial activation in the striatum is evident at autopsy in CLN2 patients (25), the striatum has been largely overlooked in CLN2 disease pathogenesis. Nevertheless, CLN2 patients often exhibit complex movement disorder phenotypes, including non-epileptic myoclonus, dystonia, spasticity, chorea, athetosis, and tremors (16), suggesting an underappreciated involvement of striatal pathology. A better understanding of movement disorder etiology and potential striatal involvement will be crucial for devising therapeutic approaches targeting a wide range of neurological symptoms of CLN2 disease. Our findings present the first evidence to our knowledge of CTIP2-positive MSN loss, along with pronounced astrogliosis and microglial activation in CLN2 mice. However, the cell-autonomous loss of MSNs within the CPu of Vgat-Cre; TPP1LAMP1 mice did not cause astrogliosis or microglial activation locally, suggesting that CLN2-disease-associated glial activation might also occur autonomously rather than as a secondary response to neuron loss, at least within this brain region. With TPPLAMP1 mice theoretically enabling us to introduce glia-specific TPP1 deficiency, it will be important to explore potential cell-autonomous effects of TPP1 deficiency in different types of glia and its contribution to other phenotypes in *Cln2^R207X/R207X^* mice. Additionally, while a previous study has shown a tremor phenotype in *Cln2^R207X/R207X^* mice (23), Vgat-Cre; TPP1LAMP1 mice did not exhibit apparent tremors by 25 weeks of age based on our gross observations. It is possible that Vgat-Cre; TPP1LAMP1 mice may develop such tremors and/or other movement disorder–related phenotypes at a later stage of disease. It will be important to further assess the potential impact of striatal neuron loss on movement disorders in Vgat-Cre; TPP1LAMP1 mice.

Epileptiform activity can be divided into at least 3 distinct stages: initiation, propagation, and termination (55). Vgat-Cre; TPP1LAMP1 mice did not develop spontaneous seizures, suggesting that interneuron-specific TPP1 deficiency is by itself not sufficient to initiate seizures. To further explore the potential role of TPP1-deficient interneurons in seizure susceptibility, we induced acute generalized seizures in Vgat-Cre; TPP1LAMP1 mice using PTZ, a commonly used method for both mechanistic studies and preclinical evaluation of antiepileptic drugs (56). There was no significant difference in latency to seizure onset between Vgat-Cre; TPP1LAMP1 mice and TPP1LAMP1 control mice. Although many TPP1LAMP1 mice experienced multiple shorter generalized seizures before advancing to fatal long-lasting seizures, we found that Vgat-Cre; TPP1LAMP1 mice were significantly more likely to die following a single episode of generalized long-lasting seizures. These results suggest that interneurons may play a critical role in terminating seizures rather than initiating them in CLN2 disease. Although the cellular mechanism of seizure termination remains under debate, multiple lines of evidence suggest a link between increased interneuronal activity and longer refractory periods that contributes to seizure termination in animal models (57). Therefore, while the nature of electrophysiological dysfunction of interneurons in CLN2 disease is unclear, it is reasonable to suspect that interneuron dysfunction caused by TPP1 deficiency and subsequent lysosomal dysfunction might lead to dysregulated seizure termination. Importantly, sudden death following seizures is a distinctive feature of *Cln2^R207X/R207X^* mice (24) compared with *Cln1^–/–^* mice, modeling CLN1 disease, which develop similar spontaneous seizures without experiencing seizure-related sudden death (58). Therefore, although the nature of PTZ-induced seizures may differ considerably from the nature of chronic spontaneous seizures of *Cln2^R207X/R207X^* mice, our findings provide insights into the mechanistic role of interneurons in the fatal seizure phenotypes of CLN2 disease.

Recent technological advancements in chemogenetics and optogenetics have enabled the study of causality between interneuron activity and seizures, revealing the protective contribution of interneuron activities to various seizure types (57, 59). Initially, we anticipated that hM3Dq-DREADD activation of interneurons would attenuate spontaneous seizures in *Cln2^R207X/R207X^* mice. However, our results robustly showed an opposite effect; chemogenetic activation of interneurons in *Cln2^R207X/R207X^* mice exacerbated their fatal seizure phenotype. Another study has reported that optogenetic activation of hippocampal PV- and SST-positive interneurons increases chemically induced epileptic discharges in WT mice (60). Additionally, silencing PV-positive interneurons in the primary motor cortex has been shown to reduce the duration of optogenetically induced seizures (61). Such findings suggest a bimodal role of interneuron activity in seizures, dependent on interneuron subpopulations, brain regions, seizure types, and disease status. We used *Vgat-Cre* mice to simultaneously target multiple interneuron populations in our experiments. This strategy was adopted because interneuron loss occurs to a similar extent in at least 5 different subpopulations (PV-, SST-, CR-, and CB-positive cortical interneurons and CTIP2-positive striatal MSNs) in *Cln2^R207X/R207X^* mice (24). While this choice of *Cre* driver line was a logical initial step to establish the involvement of interneurons in CLN2 disease, it will be informative to identify the individual interneuron subpopulation(s) that contribute most to seizure phenotypes in *Cln2^R207X/R207X^* mice.

Although most previous studies have utilized an acute DREADD approach to study chemically induced seizures, our experiment instead employed a chronic DREADD approach. This allowed us to monitor the long-term effects of DREADD activation of interneurons on naturally occurring spontaneous seizures in *Cln2^R207X/R207X^* mice. As such, caution should be exercised comparing our findings to those derived from acute DREADD experiments. Nevertheless, chronic DREADD studies are becoming increasingly common due to the recent developments in selective, potent, and long-acting DREADD ligands such as DCZ, and noninvasive ligand administration routes (62–64). However, there are several potential technical limitations associated with chronic DREADD studies, including tolerance and desensitization to the ligand (59). In particular, chronic DREADD activation of interneurons may cause “ionic plasticity,” a phenomenon where continuous activation of interneurons leads to a massive Cl^–^ influx, resulting in a qualitative change in GABA_A_R-mediated signaling from hyperpolarization (inhibition) to depolarization (excitation) (65). Such depolarized GABAergic activity has been observed with hM3Dq-DREADD activation of interneurons within the subiculum for 3 days that exacerbated electrographic seizures during the chronic phase of chemically induced epilepsy in *Vgat-Cre* mice (66), suggesting a proconvulsant effect of ionic plasticity in chronically activated interneurons. This is in line with our unexpected observation of seizures provoked by hM3Dq-DREADD–mediated activation in *Vgat-Cre; WT* mice. Nevertheless, this hM3Dq-DREADD–mediated activation did not have as severe consequences as in Vgat-Cre; TPP1LAMP1 mice, all of which died immediately after an activation-induced seizure. Therefore, a functional conversion of interneurons from inhibition to excitation might explain the seizure exacerbation in *Cln2^R207X/R207X^* mice upon chronic DREADD activation of interneurons, and future electrophysiological studies would provide more mechanistic insights into this possibility.

Our results may provide mechanistic insights into clinical management of epilepsy in CLN2 disease. Seizure control in patients with CLN2 disease typically requires multiple antiseizure medications. However, several of these, such as carbamazepine, gabapentin, phenytoin, and vigabatrin, have been reported to exacerbate myoclonic seizures (13). Notably, some of these drugs, particularly gabapentin and vigabatrin, exert their effects by modulating GABAergic signaling. While our study focused on tonic-clonic seizures, the predominant seizure type observed in *Cln2^R207X/R207X^* mice, our findings may offer a potential explanation for these paradoxical drug responses. Such mechanistic insights could potentially help clinicians make more informed decisions regarding antiseizure management in CLN2 patients.

Although localized activation of both microglia and astrocytes is widely used as a measure of the efficacy of experimental therapies across many NCLs, its pathological role in neurodegeneration remains unclear (37). Despite the proconvulsant effect of hM3Dq-DREADD activation of interneurons in *Cln2^R207X/R207X^* mice, 72-hour DREADD activation of interneurons attenuated CD68-positive microglial activation, typically interpreted as an improvement in disease phenotype. It is known that microglial processes make contact with GABAergic synapses and are involved in synaptic pruning and modulating neurotransmission (67). Emerging evidence also suggests that microglia express GABA_B_Rs and sense inhibitory neuronal activity during early developmental stages (68, 69). While little is known about microglia-interneuron interactions in the young adult brain, our findings suggest that microglial reactivity associated with CLN2 disease might be directly influenced by interneuron activity via GABA.

In contrast, hM3Dq-DREADD activation of interneurons did not alter astrogliosis, but did significantly increase GABA immunoreactivity within reactive astrocytes in the striatum. Astrocytes are both GABAergic and GABAceptive, playing an important role in GABA metabolism in the brain (41, 42). The significant increase in astrocytic GABA we observed upon hM3Dq-DREADD activation of interneurons is most likely due to the uptake of GABA released in increased amounts by stimulated interneurons. Similar increases in GABA content within reactive astrocytes are seen in various neurological conditions such as Alzheimer disease (70), kainic acid–induced epilepsy (71, 72), and stroke (73). To our knowledge, our results provide the first evidence of this occurring in any LSD model. These increases in astrocytic GABA may explain our previous findings of sustained GABA concentrations in the cortex of 3-month-old *Cln2^R207X/R207X^* mice, despite their profound loss of interneurons (24). While it remains unclear whether this elevated astrocytic GABA serves as a compensatory mechanism in response to interneuron loss in CLN2 disease, a better understanding of its pathological impact might offer an alternative therapeutic opportunity.

### Conclusions

We generated TPP1LAMP1 mice to investigate the cell-autonomous effects of conditional TPP1 deficiency. The TPP1 enzyme present in these mice has poor affinity for M6PR, rendering the small amount that is secreted incapable of cross-correction, confirming the utility of these mice as a valuable platform for generating TPP1 deficiency in specific cell types. Although interneuron-specific TPP1 deficiency did not cause more seizures, it rendered mice more susceptible to death following PTZ-induced seizures, suggesting a critical role of interneurons in seizure termination in CLN2 disease. Furthermore, chronic DREADD activation of interneurons exacerbated seizure phenotypes, underscoring the complexity of interneuron involvement in seizures associated with CLN2 disease. Additionally, our findings indicate a direct influence of interneuron activity on microglial reactivity via GABA, while increases in astrocytic GABA expression in CLN2 disease may represent a compensatory mechanism in response to interneuron loss. Our results highlight the intricate interplay between interneurons, glia, and seizures in CLN2 disease, providing valuable insights for the development of targeted therapeutic interventions.

## Methods

### Sex as a biological variable.

Mice of both sexes were included in this study, using an equal number of both male and female mice in each group for analysis.

### Mice.

*Cln2^R207X/R207X^* (The Jackson Laboratory, 030696) (23), *Vgat-Cre* (The Jackson Laboratory, 028862) (33), *Actb-Cre* (The Jackson Laboratory, 033984) (74), Ai14 (The Jackson Laboratory, 007914) (34), and WT mice were maintained on a C57BL/6J background and housed in an animal facility at the Washington University School of Medicine under a 12-hour light/dark cycle, and provided food and water ad libitum.

### Design of TPP1LAMP1 transgene and lentiviral transduction.

The 114-bp transmembrane region and cytosolic tail of LAMP1 was linked in frame to the 3′ end (C-terminus) of the 1689-bp human TPP1 cDNA (CCDS7770.1) via a 6-glycine linker to retain TPP1 within the lumen of the lysosome. The Kozak sequence was added at the 5′ end of the TPP1 cDNA, and a 30-bp c-Myc epitope tag was linked to the 3′ end (cytoplasmic tail) of the LAMP1 domain to complete the TPP1LAMP1 transgene. The TPP1LAMP1 transgene and the regular hTPP1 cDNA were each synthesized and inserted into the multiple cloning site of pLenti-III-PGK plasmid vector (Applied Biological Materials, G305). TPP1LAMP1 and TPP1 lentiviruses (LV-TPP1LAMP1 and LV-TPP1) from the plasmid were generated at the Hope Center Viral Vector Core at Washington University School of Medicine. Immortalized *Cln2*^–*/*–^** MEFs (gift from David Sleat, Rutgers New Jersey Medical School, Piscataway, New Jersey) were transduced with either LV-TPP1LAMP1 or LV-TPP1. Selection was performed by adding 3 μg/mL puromycin to the media 96 hours after transduction and continued for 1 week.

### Tissue culture.

WT, *Cln2*^–*/*–^**, and LV-TPP1– or LV-TPP1LAMP1–transduced *Cln2*^–*/*–^** MEFs were cultured in high-glucose DMEM (Gibco, 19965) supplemented with 15% fetal bovine serum (FBS) (Gibco, 10-437-028) and 1% penicillin-streptomycin (Sigma-Aldrich, P0781) on 6-well plates. When cells reached 70%–80% confluence, the media were replaced, and both cells and conditioned media were harvested 48 hours later for TPP1 activity assays. For the coculture experiments, WT, *Cln2*^–*/*–^**, and LV-TPP1– or LV-TPP1LAMP1–transduced *Cln2*^–*/*–^** MEFs were seeded onto 6-well plates at approximately 20% confluence. After 24 hours, new *Cln2*^–*/*–^** MEFs were placed on 24-mm Transwell inserts (Corning, 3450) and coincubated with preseeded MEFs. After 72 hours, *Cln2*^–*/*–^** MEFs from the inserts were harvested for the TPP1 activity assays.

### TPP1 enzyme activity assays.

TPP1 enzyme activity in samples was measured using fluorometric assays, as described previously (23, 24). See Supplemental Methods for further details.

### Generation of TPP1LAMP1-transgenic mice.

Using the pLenti-III-PGK-TPP1LAMP as a template, a poly(A) tail signal was added downstream of the c-Myc domain, and the sequence from the PGK promoter to the poly(A) tail was flanked by *loxP* loci and mouse *Rosa26* homology arms, which was subsequently inserted into the inverted terminal repeats of the AAV2 vector (AAV2-TPP1LAMP1). Transgenic mice were generated by a combination of transduction of the AAV2-TPP1LAMP1 donor vector and electroporation of CRISPR/Cas9 and the *Rosa26*-targeting guide RNA into *Cln2^R207X/WT^* embryos on the C57BL/6J background. Stable integration of the transgene was confirmed by PCR targeting both 5′ and 3′ junctions in the tail DNA. Founder mice were crossed with *Cln2^R207X/R207X^* mice to obtain *TPP1LAMP1^fl/–^*; *Cln2^R207X/R207X^* mice (F1), and F1 mice were crossed with each other to obtain *TPP1LAMP1^fl/fl^*; *Cln2^R207X/R207X^* mice (= TPP1LAMP1 mice, F2). Homozygosity and heterozygosity of the TPP1LAMP1 allele was determined by PCR targeting the mouse *Rosa26* sequence outside the transgene insertion. See Supplemental Methods for the information on PCR primers.

### CI-M6PR affinity assays.

CI-M6PR assays were performed as previously described (29, 75). Briefly, soluble CI-M6PR was purified from FBS and covalently conjugated to cyanogen bromide–activated Sepharose 4B (Sigma-Aldrich). Total protein extracts from brains of WT and TPP1LAMP1 mice were diluted with buffer A (50 mM sodium acetate, pH 4.0, and 1% Triton X-100) and incubated with the CI-M6PR beads at 4°C for 2 hours. After incubation, the beads were collected, washed twice with PBS and 1% Triton X-100, washed once with 0.1 M sodium acetate buffer, and assayed for TPP1 activity. Affinities for CI-M6PR are reported as the percentage of the starting enzyme recovered on the beads.

### Immunohistochemistry and imaging.

Processing of forebrains were performed, as described previously (76–78). See Supplemental Methods for further details. A 1-in-12 series of 40 μm coronal forebrain sections from each mouse were stained using on on-slide immunofluorescence protocol as previously described (79, 80). See Supplemental Methods for further details including primary and secondary antibodies. To visualize GABAergic interneurons for stereological counts, a 1-in-6 series of coronal sections were stained using a free-floating immunoperoxidase protocol (76, 77). See Supplemental Methods for further details including used antibodies. All images were taken on a Zeiss AxioImager Z1 microscope with StereoInvestigator (MBF Bioscience) software or a Zeiss LSM 880 confocal laser scanning microscope with Airyscan and ZEN 2 (blue edition, Zeiss) software.

### Quantitative thresholding image analysis.

To quantify storage material accumulation represented by SCMAS immunoreactivity and glial activation represented by GFAP-positive astrocytes and CD68-positive microglia, semiautomated thresholding image analysis was performed as described previously (79, 80). This involved collecting slide-scanned images at ×10 magnification (Zeiss Axio Scan Z1 fluorescence slide scanner) from each animal. Contours of appropriate anatomical regions were then drawn, and images were subsequently analyzed with Image-Pro Premier (Media Cybernetics), using an appropriate threshold that selected the foreground immunoreactivity above the background. All thresholding data (GFAP, CD68, and SCMAS) are expressed as the percentage of area within each anatomically defined region of interest that contained immunoreactivity above the set threshold for that antigen (“% immunoreactivity”).

### Colocalization image analysis.

To quantify the extent of overlap between GFAP (Alexa Fluor 488) and GABA (Alexa Flour 680) immunoreactivity, colocalization analysis was performed using ZEN 2 (blue edition, Zeiss) software on the confocal images taken with a ×20 objective. Briefly, *Z*-stack images covering the entire thickness of the section were processed using the maximum intensity projection function. The whole area of the images was analyzed using colocalization tools with appropriate thresholds set to distinguish foreground immunoreactivity from background for both channels. Three images were randomly selected per brain region per animal, and the average of the colocalization coefficients (area positive for both GFAP and GABA divided by the area positive for GFAP) is reported as results.

### Stereological counts of interneuron number.

Unbiased design-based optical fractionator counts of GABAergic interneurons were performed using StereoInvestigator (MBF Bioscience) in a 1-in-6 series of forebrain hemisections, as described previously (76, 78, 79). Neurons expressing PV, SST, and COUP-TF–interacting protein 2 (CTIP2) with clearly identifiable immunoreactivity were counted using the optical fractionator method with the following sampling scheme and objective: PV and SST in the S1BF, counting frame 180 × 200 μm, sampling grid 500 × 500 μm, objective 40×/0.95 Korr; CTIP2 in CPu, counting frame 100 × 10 μm, sampling grid 1000 × 1000 μm, objective 63× oil (NA 1.4).

### Total RNA extraction, cDNA synthesis, and PCR.

Total RNA was extracted from liver homogenates from TPP1LAMP1 mice or HEK293 cell pellets (control), and purified using TRIzol (Thermo Fisher Scientific), as previously described (81). Subsequently, cDNA was synthesized using Random Primers (Invitrogen) and SuperScript II Reverse Transcriptase (Invitrogen) according to the manufacturer’s protocol. The presence of hTPP1 and TPP1LAMP1 transcripts was confirmed by PCR on cDNA using a forward primer targeting hTPP1 in conjunction with either a reverse primer targeting hTPP1 or a reverse primer targeting the LAMP1 domain. See Supplemental Methods for primer sequences.

### Quantitative gait analysis.

The CatWalk XT gait analysis system (Noldus Information Technology) was used to study gait performance at monthly intervals, as described previously (79, 82). See Supplemental Methods for further details.

### EEG monitoring.

Experimental mice underwent continuous video EEG monitoring starting at 11 weeks, using standard methods for implanting epidural electrodes and EEG recording under isoflurane anesthesia, as previously described (83–85). Continuous bilateral cortical video-EEG signals starting at 11 weeks were acquired using a referential montage using Stellate or LabChart (AdInstruments) acquisition software and amplifiers until *Cln2^R207X^* mice died. See Supplemental Methods for further details.

### Seizure induction.

PTZ (Sigma-Aldrich) dissolved in PBS was injected intraperitoneally (75 mg/kg) into 25-week-old TPP1LAMP1 or Vgat-Cre; TPP1LAMP1 mice, as previously described (86). Mice were then individually placed into cages for observation via video recordings until death. Generalized tonic-clonic seizure were characterized by sudden loss of upright with diffuse tonic posturing followed by clonic shaking. The minimal interval of 30 seconds between seizures was implemented for analysis.

### Chronic DREADD studies.

Intracerebroventricular injections of 5 μL of either AAV9-hSyn-DIO-hM3Dq-mCherry (Addgene, 44361-AAV9) (87) or AAV9-hSyn-DIO-mCherry (Addgene, 50459-AAV9) vector at a concentration of 5 × 10^10^ gc per mouse were performed in P1 or P2 neonatal *Cln2^R207X/R207X^* mice. For EEG monitoring, administration of 10 μg/mL DCZ (Hello Bio, HB9126) via drinking water was initiated from 11 weeks (78 days) of age. DCZ-containing drinking water was refreshed every 3 or 4 days. For histological studies, micropipette-guided DCZ administration was used, as previously described (40). Briefly, animals at 11 weeks (78 days) of age were trained to drink 2 mL/kg of the 40% condensed milk solution dissolved in PBS from a single-channel P200 micropipette over 3 consecutive days, with 1 training session per day. From day 4 onwards, mice received daily doses of 500 μg/kg of DCZ dissolved in the same volume of 40% condensed milk. Mice were euthanized 24 hours after the third DCZ administration (96 hours after the first DCZ administration) for histological analysis. This dose of DCZ was determined based on our pilot study involving WT mice expressing hM3Dq-DREADDs, where doses ranging from 1 to 100 μg/mL (in drinking water) and from 10 to 1000 μg/kg/dose (in condensed milk) were assessed for increased c-Fos expression as a marker of neuronal activation.

### Statistics.

All statistical analyses were performed using Prism version 9.1.0 for MacOS (GraphPad Software). Unpaired, 2-tailed *t* tests were used for comparison between 2 groups based on distribution of data. A 1-way ANOVA with post hoc Bonferroni’s correction was used for comparison between 3 groups or more. Log-rank (Mantel-Cox) test was used for the onset of spontaneous seizures and survival studies. A *P* value of 0.05 or less was considered significant.

### Study approval.

There were no human subjects in this study. All animal procedures were performed in accordance with the NIH *Guide for the Care and Use of Laboratory Animals* (National Academies Press, 2011) under protocols 2018-0215, 2021-0292 and 24-0232 approved by the Institutional Animal Care and Use Committee (IACUC) at Washington University School of Medicine in St. Louis, Missouri, USA.

### Data availability.

All data discussed in the paper are contained in the Supporting Data Values file. All materials generated in this study, including mouse models, are available upon request.

## Author contributions

JDC, KT, MSS, MW, PID, and EBH conceived and designed the study and interpreted its results. KT carried out the pathology experiments, tissue culture experiments, viral vector injections into mice, RT-PCR, gait analysis, and statistical analysis on results from all experiments. NRR performed EEG recording and analysis. EME and LLW assisted with mouse genotyping and husbandry. SHW assisted with immunohistochemistry. HRN and MSR assisted in designing the TPP1LAMP1 transgene and generation of plasmid DNA. SQL performed TPP1 activity assays. BD designed and assisted CI-M6PR affinity assays. KT and JDC wrote the manuscript with input from all the authors. All authors read and approved the final manuscript.

## Supplementary Material

Supplemental data

Unedited blot and gel images

Supporting data values

## Version 1 08/21/2025

In-Press Preview

## Version 2 10/08/2025

Electronic publication
