# Supplementary figures and images for: GABAergic interneurons contribute to the fatal seizure phenotype of CLN2 disease mice
Source: JCI Insight. 2025 Aug 21;10(19):e184487. doi: 10.1172/jci.insight.184487 (PMC12513480; doi:10.1172/jci.insight.184487)

Full unedited gel for Figure S2A

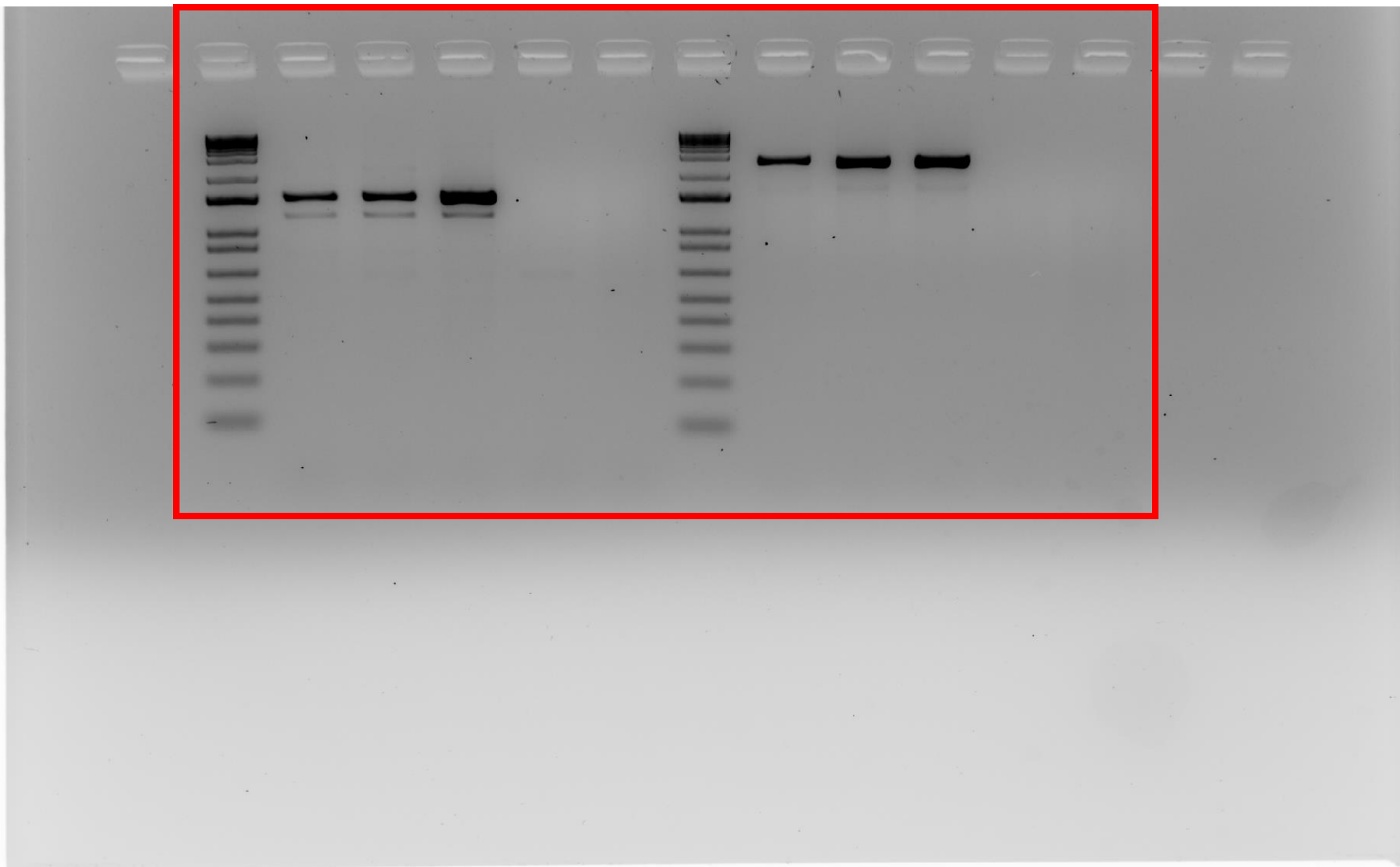

## Full unedited gel for Figure S2B

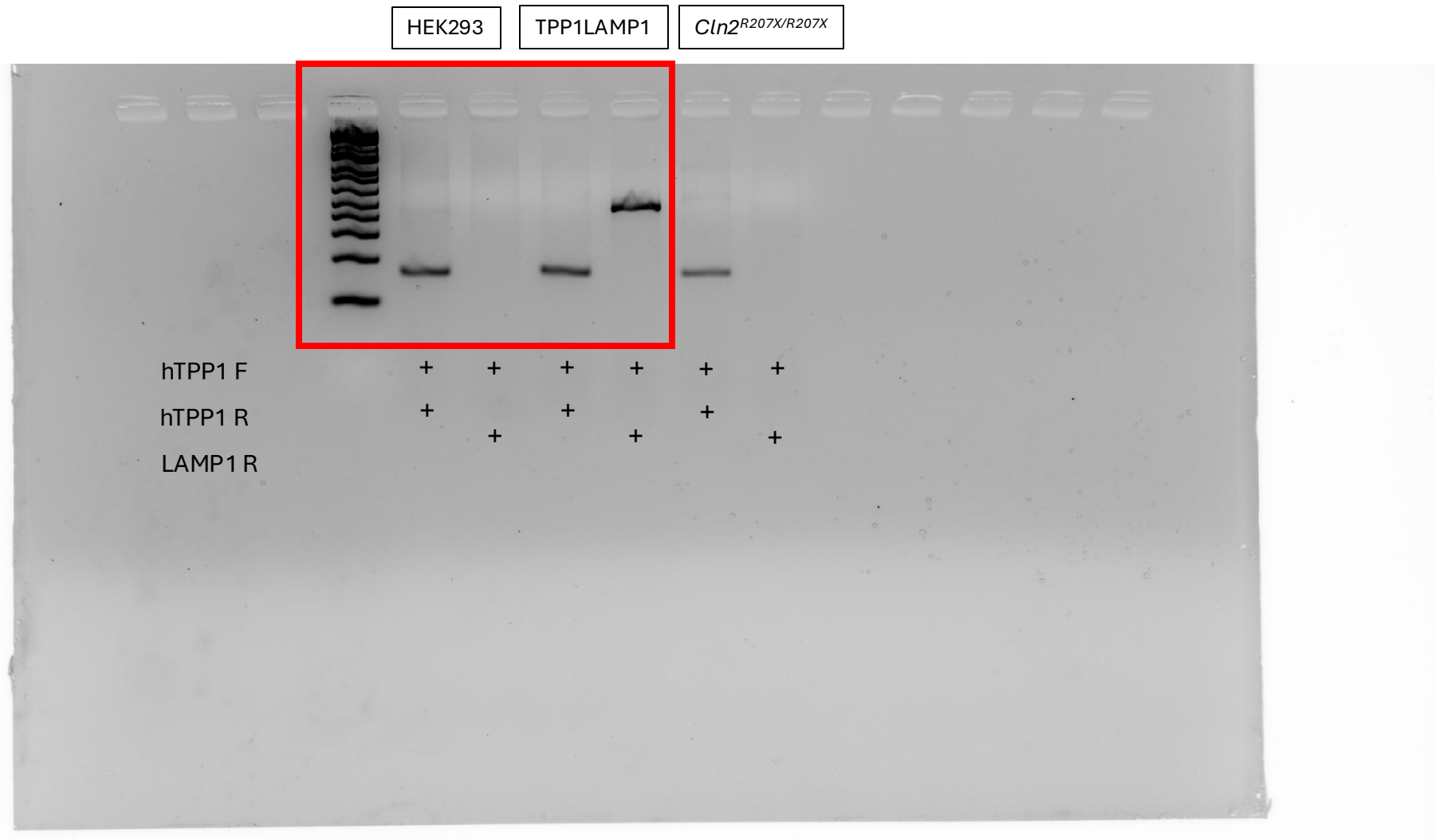

Full unedited gel for Figure S2C

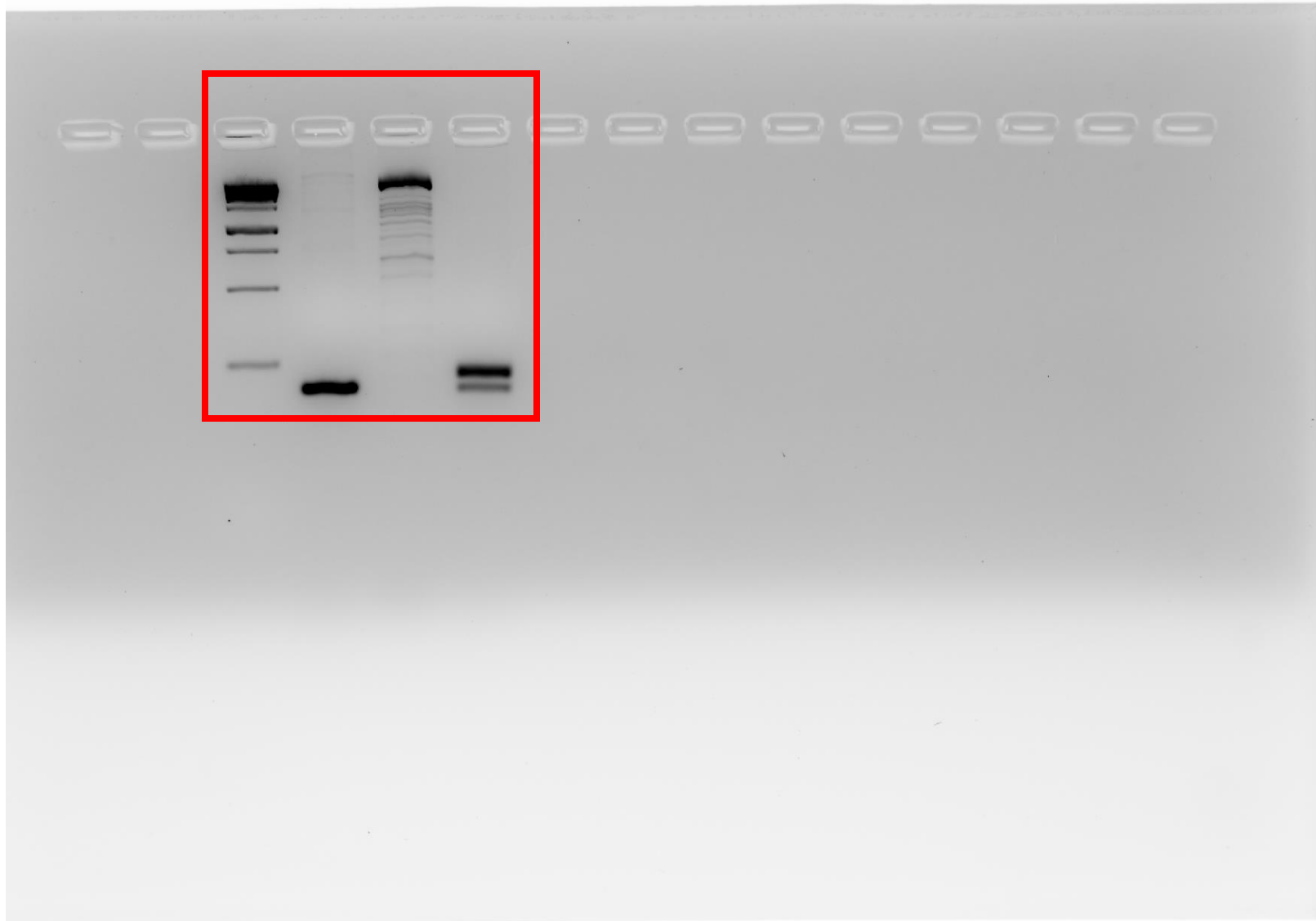

Supplement: Unedited blot and gel images [file jciinsight-10-184487-s009.pdf]
